# Relationship Between Systemic Immune‐Inflammation Index and In‐Hospital Mortality in Sepsis Combined With Chronic Obstructive Pulmonary Disease Modified by Mechanical Ventilation

**DOI:** 10.1111/crj.70122

**Published:** 2025-09-24

**Authors:** Ren‐wei Zhang, Meng‐jiao Ye

**Affiliations:** ^1^ Department of Respiratory and Critical Care Medicine Zhejiang Tiantai County Hospital of Traditional Chinese Medicine Zhejiang China

**Keywords:** COPD, in‐hospital mortality, mechanical ventilation, sepsis, SII

## Abstract

**Introduction:**

This retrospective cross‐sectional study examined the correlation between Systemic Immune‐Inflammation Index (SII) and in‐hospital mortality in patients with sepsis combined with chronic obstructive pulmonary disease (COPD) and explored the modifying effect of mechanical ventilation on this relationship.

**Methods:**

Logistic regression models were employed to explore the correlation between log SII and in‐hospital mortality. The receiver operating characteristic curve and decision curve analysis were used to examine the predictive value of log SII for in‐hospital mortality. Generalized linear regression analysis, logistic regression analysis, and restricted cubic spline were used to explore the associations among log SII, in‐hospital mortality, and mechanical ventilation states.

**Results:**

A total of 1058 patients were enrolled. Log SII was an independent risk factor for in‐hospital mortality in patients with sepsis combined with COPD (odds ratios for Model 1, Model 2, Model 3, and Model 4 were 3.116, 2.847, 2.244, and 3.495, respectively; *p* < 0.005; log SII as an optimal‐threshold categorical variable). Additionally, mechanical ventilation was closely related to log SII (*p* < 0.05). There was a stronger correlation between log SII and in‐hospital mortality of patients who received mechanical ventilation, especially those with invasive mechanical ventilation (*p* < 0.05).

**Conclusion:**

An elevated SII independently predicts elevated in‐hospital mortality risk in sepsis–COPD patients. This association is strongly intensified by mechanical ventilation, particularly the invasive mode. SII serves as a valuable biomarker for risk stratification in this vulnerable population.

## Graphical abstract

Optimal cutoff value for log SII. Abbreviation: SII: Systemic Immune‐Inflammation Index.

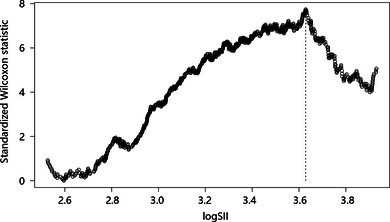

## 1 Introduction

Sepsis is a syndrome of physiologic, pathologic, and biochemical abnormalities caused by dysregulation of the host's response to infection, resulting in life‐threatening organ dysfunction [1], which has a high morbidity and mortality [2]. It is reported that there are 48.9 million sepsis cases and 11 million sepsis‐related deaths worldwide, accounting for 20% of the total global deaths [3]. Chronic obstructive pulmonary disease (COPD) is a common lung disease in clinics, which is characterized by limited airflow and dyspnea [4] COPD is one of the most common comorbidities among sepsis patients [5]. An analysis from the Medical Information Mart for Intensive Care IV (MIMIC‐IV) demonstrated significantly higher 28‐day mortality among sepsis patients with comorbid COPD compared to those without COPD [6]. Therefore, improving the prognosis of sepsis combined with COPD remains a major challenge and requires more effective treatment strategies.

The Systemic Immune‐Inflammation Index (SII) has emerged as a novel inflammatory biomarker with enhanced prognostic utility for inflammatory disorders [7]. Calculated as platelet count × neutrophil count/lymphocyte count, this composite metric integrates three key hematological parameters to holistically represent systemic inflammation status and immune homeostasis [8]. Such integration confers SII superior predictive capability relative to conventional inflammatory markers [9]. A meta‐analysis included 19 studies with 12,505 patients with urothelial carcinoma and presented that SII elevation was related to worse overall survival and cancer‐specific survival [9]. By using the large cohort from the National Health and Nutrition Examination Survey, Shi et al. [10] exhibited that the increased SII levels had a near‐linear relationship with low muscle mass. Besides, Ye et al. identified SII as a prognostic biomarker for all‐cause mortality in COPD, with mortality risk escalating proportionally to SII levels [11]. Nevertheless, no study has yet explored the association of SII with prognosis in sepsis combined with COPD.

Mechanical ventilation is a form of ventilation that utilizes a mechanical device to replace, control, or alter voluntary breathing movements [12]. Mechanical ventilation serves as a critical supportive intervention not only for managing acute exacerbations of COPD [13], but also for maintaining adequate oxygenation for sepsis [14]. However, mechanical ventilation can induce inflammatory reactions [15]. A prospective case–control study revealed that mechanically ventilated patients exhibited elevated inflammatory cell counts, increased tumor necrosis factor α (TNF‐α) concentrations, and reduced protein levels in the bronchi compared to non‐mechanically ventilated controls [16]. Therefore, this study conducted a retrospective cross‐sectional study based on the MIMIC‐IV database to analyze the correlation between SII and in‐hospital mortality in patients with sepsis combined with COPD, the correlation between mechanical ventilation and SII, and whether the correlation between SII and in‐hospital mortality was modified by mechanical ventilation therapy.

## 2 Methods

### 2.1 Data Source

Patient data were extracted from the MIMIC‐IV database 2008–2019. This publicly available critical care database, developed by the Computational Physiology Laboratory of the Massachusetts Institute of Technology, Beth Israel Deaconess Medical Center at Harvard Medical School, and Philips Healthcare, contains de‐identified clinical data from 2008 to 2019. The collection of patient information and creation of the research resource was reviewed by the Institutional Review Board at the Beth Israel Deaconess Medical Center, which granted a waiver of informed consent and approved the data sharing initiative.

### 2.2 Study Population

Inclusion criteria: aged 18 to 80 years old; diagnosed with sepsis combined with COPD. Sepsis was diagnosed according to the Sepsis 3.0 diagnostic criteria: with infection and Sequential Organ Failure Assessment (SOFA) score ≥ 2 points [17]; COPD diagnosis used *ICD‐9* codes 1144, 4162, 4168, and 4169, and *ICD‐10* codes B381, B391, B401, I2782, J811, and J953 (*n* = 5199).

Exclusion criteria: Samples lacked data on neutrophils, lymphocytes, and platelets for SII calculation (4141 excluded and 1058 remaining).

Sample size calculation: G*Power (version 3.1.9.7) was used to estimate the sample size for Cohen's d effect size index of 0.8, *α* of 0.05, power of 0.95, two‐tailed, yielding a required sample size of 88. The final cohort comprised 1058 patients, substantially exceeding the minimum sample size required for robust statistical power.

### 2.3 Data Extraction

This study retrieved demographic indicators, treatment methods, scoring indicators, vital signs, and laboratory indicators. The demographic indicators included age and sex. The treatment methods included continuous renal replacement therapy (CRRT), vasopressor use, mechanical ventilation use, type of mechanical ventilation (noninvasive and invasive), antibiotic use, and steroid use. The scoring indicators included the Glasgow Coma Scale (GCS) and SOFA. The vital signs included heart rate, systolic blood pressure (SBP), diastolic blood pressure (DBP), oxygen saturation (SpO_2_), and respiratory rate. The laboratory indicators included glucose, potassium, red cell distribution width (RDW), neutrophils, lymphocytes, hematocrit, lactate, potential of hydrogen (PH), partial pressure of oxygen (PaO_2_), partial pressure of carbon dioxide (PCO_2_), total CO_2_, platelets, white blood cell (WBC), anion gap, chloride, creatinine, international normalized ratio (INR), prothrombin time (PT), red blood cell (RBC), base excess, urea nitrogen, and urine output. The baseline data were extracted within 24 h of the initial hospitalizations. For patients with multiple admissions, data from the first admission were selected, with laboratory parameters extracted from the initial assessment performed within 24 h of admission.

SII was first calculated by the following formula: SII = platelets × neutrophils/lymphocytes.

Then, log‐transformation was applied to SII values to mitigate skewness arising from wide‐ranging data magnitudes. The optimal cutoff value of log SII was ascertained using maximally selected rank statistics analysis [18].

Primary outcome: In‐hospital mortality. In‐hospital mortality was defined as death prior to discharge from the hospital.

In addition, we retrieved microbiological testing results (blood culture and urine culture) from the “microbiology events” table in the MIMIC‐IV database. Due to the small sample size, we integrated microbiological evidence from both blood and urine cultures to classify cases into two cohorts: urinary tract infection (UTI)–attributable group (*n* = 119) and a non‐UTI group (*n* = 940).

### 2.4 Statistical Analysis

R language was used for data analysis. Quantitative data that did not follow a normal distribution were described by median (P_25_−P_75_) and statistical analyses were conducted using the Mann–Whitney *U* test between the two groups. Qualitative data were described using count (percentage), and two‐group comparisons were conducted using the chi‐square test. The variance inflation factor (VIF) test was used to test the collinearity of variables. Univariate and multivariate logistic regression models were constructed to explore the correlation between log SII and in‐hospital mortality. The receiver operating characteristic (ROC) curve and decision curve analysis (DCA) were used to examine the predictive value of log SII for in‐hospital mortality. Generalized linear regression and logistic regression analyses were used to analyze the association between mechanical ventilation and log SII. Finally, a restricted cubic spline (RCS) was used to explore the correlation between log SII and in‐hospital mortality in different mechanical ventilation states. *p* < 0.05 is considered to be statistically significant.

## 3 Results

### 3.1 Patient Information

**FIGURE 1:**
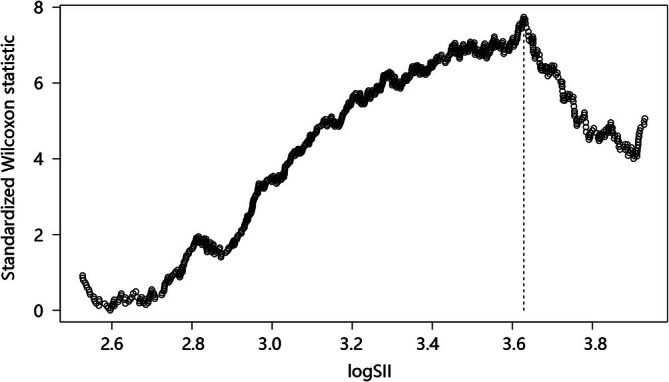
Optimal cutoff value for log SII identified through the maximally selected rank statistic. Abbreviation: SII: Systemic Immune‐Inflammation Index.

The information on patients with sepsis combined with COPD is shown in Table 1 below. A total of 1058 patients were divided into the low‐log SII group and the high‐log SII group on the basis of the optimal cutoff value (3.628, Figure 1) identified through the maximally selected rank statistic. The two groups differed in age, sex, vasopressor use, mechanical ventilation, antibiotic use, heart rate, SpO_2_, respiratory rate, glucose, RDW, neutrophils, lymphocytes, log SII, PCO_2_, total CO_2_, platelets, WBC, anion gap, chloride, INR, PT, and in‐hospital mortality. Patients in the high‐log SII group were older and had a higher proportion of females, more vasopressor users, mechanical ventilation users, and antibiotic users (*p* < 0.05). Besides, the high‐log SII group had a faster heart rate, decreased SpO_2_, and a faster respiratory rate. For laboratory indicators, elevated levels of glucose, RDW, neutrophils, log SII, PCO_2_, total CO_2_, platelets, WBC, anion gap, chloride, INR, and PT, but lower lymphocyte levels were observed in the high‐log SII group (all *p* < 0.05). In addition, the high‐log SII group accounted for a higher proportion of in‐hospital mortality cases (43.273% vs. 19.668%) (*p* < 0.001). However, there was no difference in CRRT, steroid use, UTI‐related cases, GCS, SOFA, SBP, DBP, potassium, hematocrit, lactate, pH, PaO_2_, creatinine, RBC, base excess, urea nitrogen, and urine output between the two groups (all *p* > 0.05).

**TABLE 1:** Basic information of patients.

Variable		Log SII < 3.628 (*n* = 783)	Log SII ≥ 3.628 (*n* = 275)	*p*
Age (years)		65.000 [57.000, 73.000]	68.000 [58.000, 74.000]	0.002
Sex, *n* (%)	Male	438 (55.939)	134 (48.727)	0.039
	Female	345 (44.061)	141 (51.273)	
CRRT use, *n* (%)	Yes	80 (10.217)	34 (12.364)	0.323
Vasopressor use, *n* (%)	Yes	334 (42.656)	149 (54.182)	< 0.001
Mechanical ventilation, *n* (%)	Yes	155 (19.796)	90 (32.727)	< 0.001
Antibiotic use, *n* (%)	Yes	731 (93.359)	273 (99.273)	< 0.001
Steroid use, *n* (%)	Yes	22 (2.810)	4 (1.455)	0.212
UTI‐attributable, *n* (%)	Yes	94 (12.005)	25 (9.091)	0.188
GCS		15.000 [15.000, 15.000]	15.000 [15.000, 15.000]	0.258
SOFA		3.000 [2.000, 5.000]	3.000 [2.000, 4.000]	0.055
Heart rate (bpm)		70.000 [61.000, 82.000]	76.000 [67.000, 87.000]	< 0.001
SBP (mmHg)		87.000 [78.000, 96.000]	87.000 [77.000, 95.000]	0.600
DBP (mmHg)		45.000 [39.000, 50.000]	44.000 [38.000, 50.000]	0.157
SpO_2_ (%)		92.000 [89.000, 94.000]	91.000 [87.000, 94.000]	0.004
Respiratory rate (min)		12.000 [10.000, 15.000]	14.000 [11.000, 16.000]	< 0.001
Glucose (mg/dL)		102.000 [86.000, 125.000]	112.000 [92.000, 140.000]	< 0.001
Potassium (mEq/L)		3.300 [3.100, 3.600]	3.300 [3.000, 3.600]	0.978
RDW (%)		14.100 [13.200, 15.600]	14.500 [13.500, 15.900]	0.018
Neutrophils (%)		72.000 [62.100, 80.900]	80.400 [72.500, 88.200]	< 0.001
Lymphocytes (%)		8.000 [5.100, 13.400]	2.100 [1.700, 3.600]	< 0.001
Log SII		3.092 [2.777, 3.363]	3.866 [3.737, 4.013]	< 0.001
Hematocrit (%)		24.400 [21.000, 29.100]	24.000 [21.300, 28.800]	0.992
Lactate (mg)		1.300 [1.000, 1.800]	1.300 [1.000, 1.800]	0.719
PH		7.300 [7.220, 7.370]	7.300 [7.220, 7.370]	0.798
PaO_2_ (mmHg)		67.000 [43.000, 90.000]	60.000 [43.000, 82.000]	0.101
PCO_2_ (mmHg)		37.000 [32.000, 43.000]	40.000 [34.000, 46.000]	< 0.001
Total CO_2_ (mmHg)		23.000 [20.000, 26.000]	25.000 [20.000, 29.000]	0.018
Platelets (mL)		147.000 [100.000, 204.000]	248.000 [175.000, 348.000]	< 0.001
WBC (K/μL)		9.900 [6.800, 13.200]	14.400 [10.900, 20.000]	< 0.001
Anion gap (mEq/L)		12.000 [10.000, 14.000]	13.000 [11.000, 16.000]	< 0.001
Chloride (mEq/L)		102.000 [98.000, 106.000]	100.000 [96.000, 104.000]	< 0.001
Creatinine (mg/dL)		0.900 [0.700, 1.300]	0.900 [0.700, 1.700]	0.416
INR		1.200 [1.100, 1.400]	1.300 [1.100, 1.500]	0.048
PT (s)		13.300 [12.100, 15.200]	13.900 [12.100, 16.100]	0.017
RBC (m/μL)		2.710 [2.360, 3.210]	2.720 [2.370, 3.180]	0.963
Base excess (mEq/L)		−1.000 [−2.000, 0.000]	−1.000 [−1.000, 0.000]	0.101
Urea nitrogen (mg/dL)		13.000 [10.000, 20.000]	14.000 [10.000, 28.000]	0.052
Urine output (mL)		15.000 [0.000, 25.000]	15.000 [0.000, 25.000]	0.235
In‐hospital mortality, *n* (%)	Yes	154 (19.668)	119 (43.273)	< 0.001

Abbreviations: CRRT, continuous renal replacement therapy; DBP, diastolic blood pressure; GCS, Glasgow Coma Scale; INR, international normalized ratio; PaO_2_, partial pressure of oxygen; PCO_2_, partial pressure of carbon dioxide; PH, potential of hydrogen; PT, prothrombin time; RBC, red blood cell; RDW, red cell distribution width; SBP, systolic blood pressure; SII, Systemic Immune‐Inflammatory Index; SOFA, Sequential Organ Failure Assessment; SpO_2_, oxygen saturation; UTI, urinary tract infection; WBC, white blood cell.

Although the analytic cohort met the minimum sample size requirement, substantial exclusion of cases due to missing SII components necessitated comparative analysis of baseline characteristics between included and excluded subjects among sepsis patients combined with COPD. Table S1 demonstrated that included patients were significantly older (66.000 vs. 65.000 years) and had higher SOFA scores, more frequent mechanical ventilation, and elevated in‐hospital mortality (all *p* < 0.001). There was no statistical difference in gender between the two groups (*p* > 0.05). This pattern indicates that patients with disease severity are more likely to receive comprehensive inflammatory testing within 24 h of admission.

### 3.2 Correlation of Log SII With In‐Hospital Mortality in Patients With Sepsis Combined With COPD

Since neutrophils, platelets, and lymphocytes were included in the calculation of SII, the three variables were excluded from subsequent analyses to avoid collinearity. We further tested the collinearity of the significant baseline variables. With a VIF value > 2 as a threshold, PT (VIF = 100.983), INR (VIF = 100.979), total CO_2_ (VIF = 3.249), and PCO_2_ (VIF = 2.746) were eliminated, and the variables (log SII, anion gap, chloride, vasopressor, heart rate, RDW, age, SpO_2_, respiratory rate, antibiotic use, WBC, mechanical ventilation, glucose, sex) remained (Table 2).

**TABLE 2:** Collinearity analysis.

Variable	Variance inflation factor
Prothrombin time	100.983
International normalized ratio	100.979
Total CO_2_	3.249
PCO_2_	2.746
Anion gap	1.508
Chloride	1.362
Vasopressor	1.277
Heart rate	1.179
Age	1.145
RDW	1.13
Log SII	1.119
SpO_2_	1.116
Respiratory rate	1.113
Antibiotic use	1.107
Mechanical ventilation	1.095
Glucose	1.089
White blood cell	1.062
Sex	1.041

Abbreviations: PCO_2_, partial pressure of carbon dioxide; RDW, red cell distribution width; SII, Systemic Immune‐Inflammatory Index; SpO_2_, oxygen saturation.

We further used logistic regression to explore the relationship between log SII and in‐hospital mortality in patients with sepsis combined with COPD. As shown in Table 3, when setting log SII as a continuous variable, log SII was positively correlated with in‐hospital mortality (Model 1: OR = 1.419 [1.135, 1.798], *p* = 0.003; Model 2: OR = 1.326 [1.008, 1.744], *p* = 0.001; Model 3: OR = 1.265 [0.945, 1.694], *p* = 0.114; Model 4: OR = 1.585 [1.255, 2.030]). The same result was also found in the setting log SII as a categorical variable based on the optimal cutoff value (Model 1: OR = 3.116 [2.316, 4.191], *p* < 0.001; Model 2: OR = 2.847 [1.977, 4.100], *p* < 0.001; Model 3: OR = 2.244 [1.475, 3.414], *p* < 0.001; Model 4: OR = 3.495 [2.573, 4.755], *p* < 0.001). When log SII was divided into three groups based on log SII tertiles, there was an increased risk of in‐hospital mortality as log SII increased. The above results suggested that higher log SII was associated with an increased risk of in‐hospital mortality in patients with sepsis combined with COPD.

**TABLE 3:** Relationship between log SII and in‐hospital mortality in patients with sepsis combined with COPD.

	Model 1		Model 2		Model 3		Model 4	
Variable	OR (95% CI)	*p*	OR (95% CI)	*p*	OR (95% CI)	*p*	OR (95% CI)	*p*
Log SII	1.419 (1.135–1.798)	0.003	1.326 (1.008–1.744)	0.044	1.265 (0.945–1.694)	0.114	1.585 (1.255–2.030)	< 0.001
Log SII group^a^	3.116 (2.316–4.191)	< 0.001	2.847 (1.977–4.100)	< 0.001	2.244 (1.475–3.414)	< 0.001	3.495 (2.573–4.755)	< 0.001
Log SII tertiles								
(0.0000, 3.0297)	Ref.		Ref.		Ref.		Ref.	
(3.0309, 3.5250)	1.110 (0.756–1.628)	0.596	1.048 (0.651–1.690)	0.846	1.163 (0.668–2.024)	0.593	1.261 (0.849–1.873)	0.251
(3.5251, 4.6654)	2.896 (2.037–4.118)	< 0.001	2.491 (1.586–3.913)	< 0.001	3.282 (1.365–3.875)	0.002	3.432 (2.376–4.958)	< 0.001

*Note:* Model 1 without adjustment; Model 2 adjusting anion gap, chloride, WBC, glucose, RDW; Model 3 adjusting anion gap, chloride, WBC, glucose, RDW, heart rate, age, SpO_2_, respiratory rate, vasopressor, mechanical ventilation, sex; Model 4 adjusting SOFA and GCS scores.

Abbreviations: COPD, chronic obstructive pulmonary disease; OR (95% CI), odds ratio (95% confidence interval); SII, Systemic Immune‐Inflammatory Index.

^a^ Patients were divided into a low log SII group and a high log SII group based on the optimal cutoff value.

Moreover, we conducted sensitivity analyses by excluding samples without using antibiotics and excluding those using steroids, respectively. After excluding patients without antibiotic therapy, elevated log SII remained associated with in‐hospital mortality, irrespective of whether log SII was modeled as a continuous variable (OR: 1.346; 95% CI: 1.081–1.699; *p* < 0.001) or categorical variable (tertile 3 vs. tertile 1 OR: 2.832; 95% CI: 1.980–4.051; *p* < 0.001) (Table 4). After exclusion of steroid‐treated patients, the significant relationship between log SII and in‐hospital mortality remained robust (Table 4).

**TABLE 4:** Sensitivity analyses of the relationship between log SII and in‐hospital mortality.

	Excluding samples without using antibiotics (*n* = 54)		Excluding samples using steroids (*n* = 26)	
Variable	OR (95% CI)	*p*	OR (95% CI)	*p*
Log SII	1.346 (1.081–1.699)	0.010	1.387 (1.110–1.757)	0.005
Log SII group^a^	2.943 (2.184–3.968)	< 0.001	3.095 (2.294–4.175)	< 0.001
Log SII tertiles				
(0.0000, 3.0297)	Ref.		Ref.	
(3.0309, 3.5250)	1.214 (0.827–1.782)	0.323	1.115 (0.757–1.642)	0.582
(3.5251, 4.6654)	2.832 (1.980–4.051)	< 0.001	2.853 (1.999–4.071)	< 0.001

Abbreviations: OR (95% CI), odds ratio (95% confidence interval); SII, Systemic Immune‐Inflammatory Index.

^a^ Patients were divided into a low log SII group and a high log SII group based on the optimal cutoff value.

**FIGURE 2:**
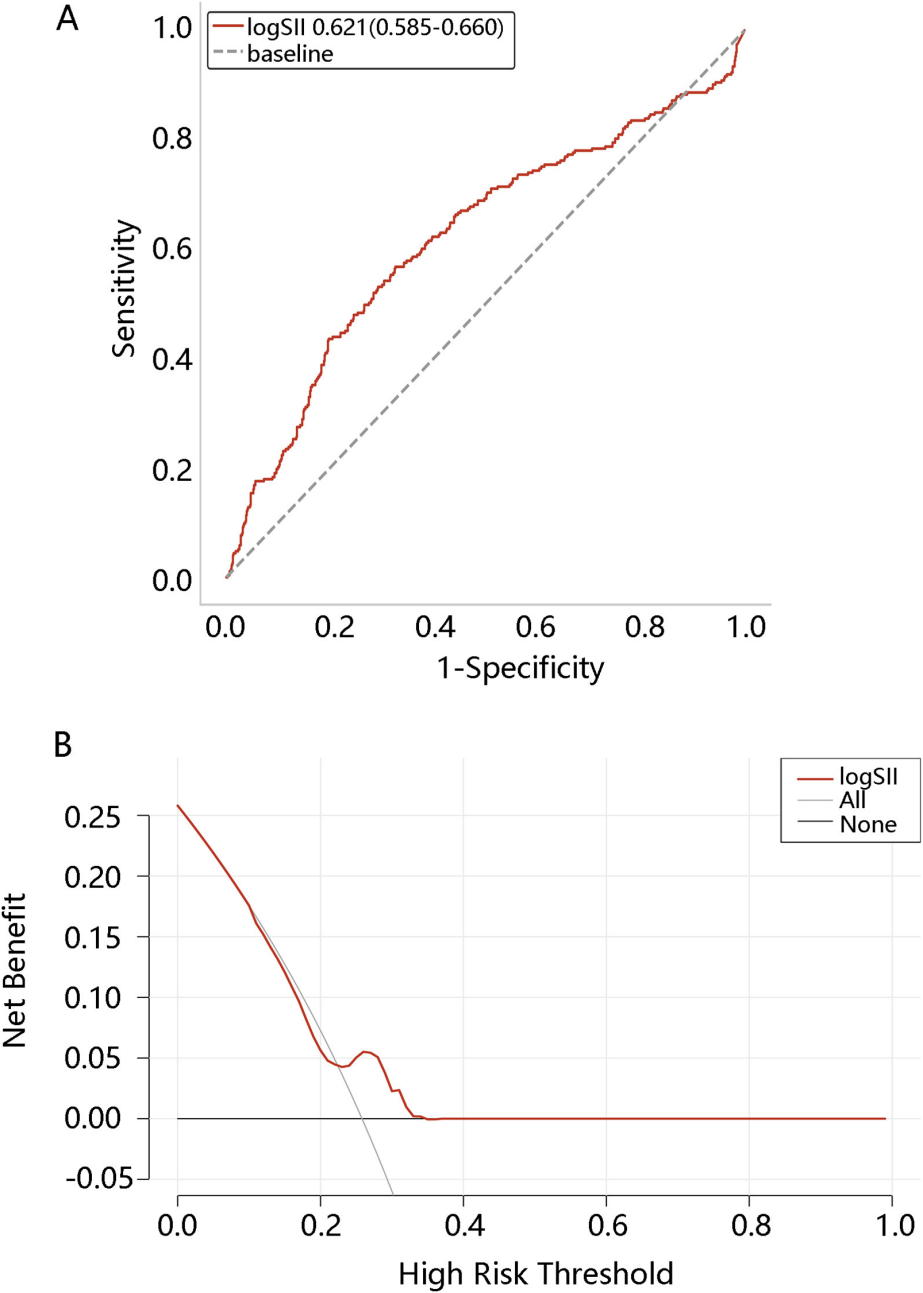
The predictive value of log SII for in‐hospital mortality in sepsis patients with chronic obstructive pulmonary disease. (A) Receiver operating characteristic curve analysis. (B) Decision curve analysis. Abbreviation: SII: Systemic Immune‐Inflammatory Index.

To study the clinical value of log SII, we further explored its predictive value by ROC and DCA analyses. As shown in Figure 2A, the area under the curve value of log SII was 0.621 (0.585–0.660). From Figure 2B, when the threshold was about 0.22 to 0.33, the clinical net benefit of log SII was higher than the treat‐none model and the treat‐all model. These suggested that log SII had good clinical value.

### 3.3 Correlation of Mechanical Ventilation With Log SII in Patients With Sepsis Combined With COPD

Since mechanical ventilation may induce inflammatory reactions [16], we further explored the correlation between mechanical ventilation and log SII in patients with sepsis combined with COPD. The results of the generalized linear regression (Table 5) showed a positive correlation between mechanical ventilation and log SII in Model 1, Model 2, and Model 3 (Model 1: *β* = 0.213 [0.117–0.310], *p* < 0.001; Model 2: *β* = 0.239 [0.133–0.344], *p* < 0.001; Model 3: *β* = 0.214 [0.095–0.332], *p* < 0.001). When log SII was treated as a categorical variable based on the optimal cutoff value, there was also a positive correlation between mechanical ventilation and log SII (Model 1: OR = 1.971 [1.450–2.680], *p* < 0.001; Model 2: OR = 2.254 [1.565–3.245], *p* < 0.001; Model 3: OR = 1.925 [1.278–2.898], *p* = 0.002). These findings indicated that mechanical ventilation was closely related to log SII.

**TABLE 5:** Association of mechanical ventilation with log SII in patients with sepsis combined with COPD.

	Model	Variable	*β*/OR (95% CI)	*p*
Log SII	Model 1	Mechanical ventilation	0.213 (0.117–0.310)	< 0.001
	Model 2	Mechanical ventilation	0.239 (0.133–0.344)	< 0.001
	Model 3	Mechanical ventilation	0.214 (0.095–0.332)	< 0.001
Log SII group^a^	Model 1	Mechanical ventilation	1.971 (1.450–2.680)	< 0.001
	Model 2	Mechanical ventilation	2.254 (1.565–3.245)	< 0.001
	Model 3	Mechanical ventilation	1.925 (1.278–2.898)	0.002

*Note:* Model 1 without adjustment; Model 2 adjusting anion gap, chloride, WBC, glucose, RDW; Model 3 adjusting anion gap, chloride, WBC, glucose, RDW, heart rate, age, SpO_2_, respiratory rate, vasopressor, sex.

Abbreviations: COPD, chronic obstructive pulmonary disease; OR (95% CI), odds ratio (95% confidence interval); SII, Systemic Immune‐Inflammatory Index.

^a^ Patients were divided into a low log SII group and a high log SII group based on the optimal cutoff value.

### 3.4 Correlation of log SII and In‐Hospital Mortality in Different Mechanical Ventilation States

**FIGURE 3:**
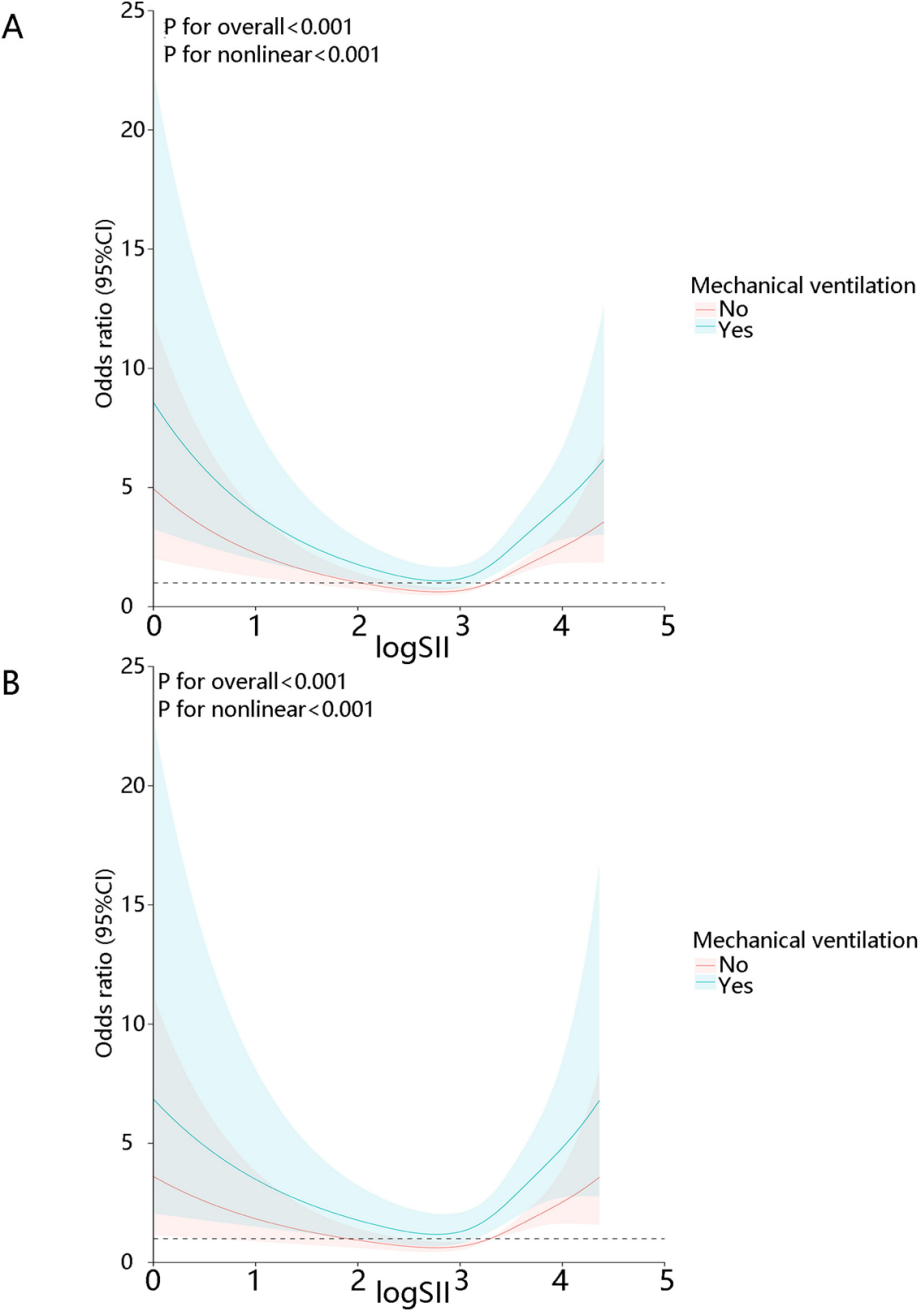
Nonlinear relationship between log SII and in‐hospital mortality stratified by mechanical ventilation status. (A) Unadjusted model. (B) Adjusted model: adjusting anion gap, chloride, WBC, glucose, RDW, heart rate, age, SpO_2_, respiratory rate, vasopressor, mechanical ventilation, and sex. The shaded bands represent a 95% confidence interval. Abbreviation: SII: Systemic Immune‐Inflammatory Index.

Our analysis demonstrated that elevated log SII was associated with in‐hospital mortality in patients with sepsis combined with COPD. Concurrently, the mechanical ventilation requirement independently correlated with higher log SII levels. Therefore, we hypothesized that the correlation between log SII and in‐hospital mortality may be modified by mechanical ventilation. Accordingly, we stratified the cohort based on mechanical ventilation status and employed RCS analysis to characterize the nonlinear association between the log SII and in‐hospital mortality. As illustrated in Figure 3A,B, the association between elevated log SII and in‐hospital mortality was significantly enhanced in mechanically ventilated patients, which persisted in both unadjusted and adjusted models: (unadjusted model: *p* for overall < 0.001, *p* for nonlinear < 0.001; adjusted model: *p* for overall < 0.001, *p* for nonlinear < 0.001).

To further investigate the association between log SII and in‐hospital mortality in patients with sepsis combined with COPD, we performed stratified logistic regression analysis based on mechanical ventilation status as well as the type of mechanical ventilation. As shown in Table 6, when setting log SII as a continuous variable, in the mechanical ventilation group, for each standard deviation increase in log SII, the risk of in‐hospital mortality increased by 86%, while in the without mechanical ventilation group, for each standard deviation increase in log SII, the risk of in‐hospital mortality increased by 19.5%. When log SII was divided into three groups based on log SII tertiles, in the mechanical ventilation group, compared with the reference group, for each standard deviation increase in log SII, the risk of in‐hospital mortality increased by 206.9% in the highest tertile group, while in the without mechanical ventilation group, that increased by 164.3%.

**TABLE 6:** Association of log SII and in‐hospital mortality in different mechanical ventilation treatment groups.

	OR (95% CI)		OR (95% CI)	
	MV group	*p*	Without the MV group	*p*
Log SII	1.860 (1.162–3.184)	0.016	1.195 (0.930–1.561)	< 0.001
Log SII group^a^	2.267 (1.323–3.882)	0.003	3.283 (2.292–4.702)	< 0.001
Log SII tertiles				
(0.0000, 3.0297)	Ref.		Ref.	
(3.0309, 3.5250)	1.959 (0.980–3.913)	0.057	0.970 (0.610–1.543)	0.897
(3.5251, 4.6654)	3.069 (1.549–6.079)	0.001	2.643 (1.745–4.004)	< 0.001
	Noninvasive MV group		Invasive MV group	
Log SII	2.721 (0.998–11.460)	0.117	1.709 (1.012–3.118)	0.062
Log SII group^a^	3.704 (1.028–13.346)	0.045	2.045 (1.126–3.713)	0.019
Log SII tertiles				
(0.0000, 3.0297)	Ref.		Ref.	
(3.0309, 3.5250)	0.800 (0.170–3.767)	0.778	2.869 (1.294–6.360)	0.009
(3.5251, 4.6654)	2.400 (0.524–10.992)	0.259	3.660 (1.659–8.074)	0.001

Abbreviations: MV, mechanical ventilation; OR (95% CI), odds ratio (95% confidence interval); SII, Systemic Immune‐Inflammatory Index.

^a^ Patients were divided into a low log SII group and a high log SII group based on the optimal cutoff value.

Moreover, we explored their association according to mechanical ventilation type. In the noninvasive ventilation subgroup, the association of log SII with in‐hospital mortality demonstrated different statistical significance by setting log SII as a continuous variable, optimal‐threshold categorical variable, and tertiles. Statistical significance was achieved only when log SII was set as an optimal‐threshold categorical variable, but the *p* value was still close to 0.05 (OR [95% CI]: 3.704 [1.028–13.346]; *p* = 0.045). Conversely, in the invasive ventilation group, the second (OR [95% CI]: 2.869 [1.294–6.360]; *p* = 0.009) and third (OR [95% CI]: 3.660 [1.659–8.074]; *p* = 0.001) tertile groups still possessed an increased in‐hospital risk compared to the first tertile group (Table 6). These results suggested that mechanical ventilation strengthens the association between log SII and in‐hospital mortality compared to non‐ventilated patients. Moreover, invasive mechanical ventilation further potentiates this relationship relative to the noninvasive ventilation group.

### 3.5 Subgroup Analysis by Infection Source

Moreover, we conducted subgroup analysis according to infection source. In the UTI‐related group, irrespective of whether the log SII was coded as a continuous variable, an optimal‐threshold categorical variable, or a tertile‐based variable, no significant association with in‐hospital mortality was observed (all *p* > 0.05). In the group without UTI‐related infections, regardless of categorization as a continuous variable (OR [95% CI]: 1.377 [1.079–1.758]; *p* = 0.010), an optimal‐threshold categorical variable (OR [95% CI]: 3.338 [2.441–4.566]; *p* < 0.001), or a tertile‐based categorical variable (compared to the lowest tertile group, the highest tertile group: OR [95% CI]: 2.937 [2.025–4.260]; *p* < 0.001), elevated log SII exhibited significant associations with in‐hospital mortality (Table 7).

**TABLE 7:** Association of log SII and in‐hospital mortality in different UTI‐related groups.

	OR (95% CI)		OR (95% CI)	
	UTI‐attributable group	*p*	Non‐UTI group	*p*
Log SII	1.758 (0.868–3.559)	0.117	1.377 (1.079–1.758)	0.010
Log SII group^a^	1.636 (0.620–4.317)	0.320	3.338 (2.441–4.566)	< 0.001
Log SII tertiles				
(0.0000, 3.0297)	Ref.		Ref.	
(3.0309, 3.5250)	1.403 (0.444–4.433)	0.564	1.098 (0.732–1.649)	0.651
(3.5251, 4.6654)	2.327 (0.774–6.993)	0.132	2.937 (2.025–4.260)	< 0.001

Abbreviations: OR (95% CI), odds ratio (95% confidence interval); SII, Systemic Immune‐Inflammatory Index; UTI, urinary tract infection.

^a^ Patients were divided into a low log SII group and a high log SII group based on the optimal cutoff value.

### 3.6 Association of Log SII and In‐Hospital Mortality in Sepsis Patients Without COPD

To validate the generalizability of our results, the association of log SII and in‐hospital mortality in sepsis patients without COPD was explored. When taking log SII as a continuous variable, log SII was linked to in‐hospital mortality with OR (95% CI) = 1.598 (1.344–1.905) (*p* < 0.001) in unadjusted Model 1. After adjusting for various covariates, this significant relationship remained robust (Model 2: OR [95% CI] = 1.358 [1.102–1.674]; Model 3: OR [95% CI] = 1.180 [0.928–1.500]; Model 4: OR [95% CI] = 1.794 [1.498–2.150]). When log SII was divided into three tertiles, compared to the lowest tertile, the highest tertile exhibited a higher in‐hospital mortality risk both in unadjusted and adjusted models (all *p* < 0.05) (Table S2). These findings demonstrate that log SII retains its significant association with in‐hospital mortality in sepsis patients without comorbid COPD, confirming its prognostic validity across heterogeneous sepsis populations.

## 4 Discussion

This study conducted a retrospective cross‐sectional survey and found that (1) log SII was positively correlated with in‐hospital mortality in patients with sepsis combined with COPD; (2) there was a positive correlation between mechanical ventilation and log SII; (3) the use of mechanical ventilation will strengthen the correlation between log SII and in‐hospital mortality; moreover, invasive mechanical ventilation possesses a more pronounced relationship relative to the noninvasive ventilation group.

First, log SII levels were significantly related to an increase in‐hospital mortality risk across four different logistic regression models. Elevated SII values were characterized by increased platelet and neutrophil counts concurrent with decreased lymphocyte levels, reflecting a pro‐inflammatory hematological profile. Possible mechanisms under the positive relationship between log SII and in‐hospital mortality may be as follows: (1) activated neutrophils release neutrophil extracellular traps (NETs), which are intended to capture pathogens [19]. However, NETs are overproduced in sepsis, which may induce endothelial cells to shift toward a pro‐inflammatory phenotype, leading to organ dysfunction [20]. The lungs of COPD patients already have chronic inflammation, and the release of NETs will further aggravate lung damage. In addition, massive infiltration of neutrophils releases excessive pro‐inflammatory mediators [21]. When sepsis is combined with COPD, this “inflammatory storm” becomes particularly intense, leading to systemic vasodilation, capillary leakage, tissue edema, and multiple organ failure, directly increasing the risk of death. (2) Lymphocytes, an indicator of immunity, have been reported to decrease during inflammation due to an increase in apoptosis [22]. In patients with advanced and severe sepsis, a large number of lymphocytes (especially T cells, B cells, and natural killer cells) undergo apoptosis, leading to a significant decline in the host's ability to recognize and clear antigens [23]. COPD may also have a certain degree of systemic inflammation and lymphocyte immune dysfunction [24]. The immunosuppressive state makes patients highly prone to secondary hospital‐acquired infections, thereby increasing the risk of death. (3) During sepsis and inflammation stimuli, platelets are highly activated. Activated platelets form microthrombi by interacting with neutrophils, monocytes, and endothelial cells, leading to microcirculation disorders in tissues and organs, ischemia, hypoxia, and organ failure [25, 26]. Patients with COPD often have a hypercoagulable state and impaired endothelial function, which further exacerbates this process. Besides SII, there are stable and easy‐to‐obtain blood biomarkers such as systemic inflammation response index [27] and aggregate index of systemic inflammation [28]. We conducted a preliminary analysis to compare the clinical value of SII, neutrophil–lymphocyte ratio, and platelet–lymphocyte ratio. The results showed that the efficacy of these three indicators in predicting in‐hospital mortality is comparable (Table S3). These suggest that we can further explore the relationship between these indicators and the prognosis of patients with sepsis combined with COPD in the follow‐up study and determine a better prediction model.

Further, we found a positive correlation between mechanical ventilation and SII. Mechanical ventilation is divided into invasive and noninvasive types. Fernandes et al. found that both invasive and noninvasive ventilation patients had a higher SII than the healthy population, and that invasive mechanical ventilation patients had a higher SII than those with noninvasive ventilation [29]. Preoperative SII predicts the need for postoperative mechanical ventilation [30] as well as prolonged mechanical ventilation [31]. Similarly, we found a positive relationship between mechanical ventilation and SII, and the use of mechanical ventilation will strengthen the correlation between log SII and hospital death in septic patients with COPD. Additionally, invasive mechanical ventilation further potentiates this relationship compared to the noninvasive ventilation group. Mechanical ventilation, especially when it is invasive, can result in improper tidal volume and pressure, which will impose mechanical stress on the already fragile (damaged due to sepsis and COPD) lung tissue. This stress will activate immune cells within the lungs (such as alveolar macrophages), causing them to release large amounts of pro‐inflammatory cytokines into the systemic circulation [32].

Our findings establish SII as a robust, readily available prognostic biomarker for in‐hospital mortality risk stratification in sepsis–COPD patients. The effect modification by invasive mechanical ventilation underscores that this high‐inflammatory, high‐thrombotic, and immunosuppressed phenotype is exceptionally vulnerable to secondary injury. The critical insight from our study lies in the disconnect between treatment and outcome: despite no difference in steroid use between the two SII groups (which may be due to fewer steroid users), mortality diverged significantly. The significant association of SII and in‐hospital mortality persisted after excluding steroid‐treated patients, highlighting a profound unmet therapeutic need in the high‐SII population. We therefore posit that SII transcends prognostication. It identifies a targetable endotype that may derive superior benefit from a precision medicine approach. We hypothesize that high‐SII patients, particularly those requiring invasive ventilation, represent the ideal candidates for future clinical trials testing immunomodulatory therapies (e.g., corticosteroids or interleukin‐6 antagonists).

For limitations, the duration of mechanical ventilation was not included in the model due to a very high rate of missing data. Besides, direct metrics of COPD severity are not available in the MIMIC‐IV database. Due to the retrospective cross‐sectional study design, although we validated the results in sepsis without COPD patients to improve generalizability, prospective validation of SII as a predictive biomarker with more covariates included to guide such targeted interventions and ultimately improve outcomes in this high‐risk cohort is still needed.

## 5 Conclusions

In conclusion, an elevated SII is an independent predictor of in‐hospital mortality in patients with sepsis and COPD. This association is markedly potentiated by mechanical ventilation, with the strongest effect observed in the invasively ventilated subgroup. These findings establish SII as a crucial integrative biomarker that captures the heightened risk arising from the synergy between a patient's systemic inflammatory status and the additional insult of mechanical ventilation. Therefore, SII provides a practical tool for early risk stratification and identifies a vulnerable phenotype that warrants more intensive monitoring and personalized management strategies.

## Author Contributions

R.W.Z. contributed to the conception and design. R.W.Z. and M.J.Y. contributed to the collection and assembly of data. R.W.Z. and M.J.Y. analyzed and interpreted the data. All authors wrote and approved the final manuscript.

## Ethics Statement

The Ethics Committee of Zhejiang Tiantai County Hospital of Traditional Chinese Medicine deemed that this research is based on open‐source data, so the need for ethics approval was waived. Our study complies with the Declaration of Helsinki.

## Consent

The authors have nothing to report.

## Conflicts of Interest

The authors declare no conflicts of interest.

## Supporting information


**Table S1:** Basic information of patients between the excluded and included cohorts among sepsis patients with COPD.

**Table S2:** Relationship between log SII and in‐hospital mortality in sepsis patients without COPD.

**Table S3:** Comparison of SII and other inflammation indicators.

## Data Availability

The data that support the findings of this study are available from the corresponding author upon reasonable request.
