# Supplementary material for: Relationship Between Systemic Immune‐Inflammation Index and In‐Hospital Mortality in Sepsis Combined With Chronic Obstructive Pulmonary Disease Modified by Mechanical Ventilation
Source: Clin Respir J. 2025 Sep 24;19(9):e70122. doi: 10.1111/crj.70122 (PMC12458911; doi:10.1111/crj.70122)
Supplement: Supplementary file 1 — Table S1: Basic information of patients between the excluded and included cohorts among sepsis patients with COPD. [file CRJ-19-e70122-s002.docx]

| **Table S1** Basic information of patients between the excluded and included cohorts among sepsis patients with COPD | | | | |
| --- | --- | --- | --- | --- |
| Variable |  | Excluded cohort (n=4141) | Included cohort (n= 1058) | P |
| Age (years) | | 65.000[56.000,72.000] | 66.000[57.000,73.000] | <0.001 |
| Sex n (%) | Male | 2106(50.857) | 572(54.064) | 0.062 |
|  | Female | 2035(49.143) | 486(45.936) |  |
| Mechanical ventilation n (%) | No | 3573(86.284) | 813(76.843) | <0.001 |
|  | Yes | 568(13.716) | 245(23.157) |  |
| SOFA |  | 3.000[2.000,4.000] | 3.000[2.000,5.000] | <0.001 |
| In-hospital mortality n (%) | No | 3690(89.109) | 785(74.197) | <0.001 |
|  | Yes | 451(10.891) | 273(25.803) |  |

SOFA: Sequential Organ Failure Assessment, COPD: chronic obstructive pulmonary disease
