# Supplementary material for: Relationship Between Systemic Immune‐Inflammation Index and In‐Hospital Mortality in Sepsis Combined With Chronic Obstructive Pulmonary Disease Modified by Mechanical Ventilation
Source: Clin Respir J. 2025 Sep 24;19(9):e70122. doi: 10.1111/crj.70122 (PMC12458911; doi:10.1111/crj.70122)
Supplement: Supplementary file 2 — Table S2: Relationship between log SII and in‐hospital mortality in sepsis patients without COPD. [file CRJ-19-e70122-s001.docx]

| **Table S2** Relationship between log SII and in-hospital mortality in sepsis patients without COPD | | | | | | | | |
| --- | --- | --- | --- | --- | --- | --- | --- | --- |
|  | Molde 1 |  | Model 2 |  | Model 3 |  | Model 4 |  |
| Variable | OR (95%CI) | P | OR (95%CI) | P | OR (95%CI) | P | OR (95%CI) | P |
| Log SII | 1.598 (1.344 - 1.905) | <0.001 | 1.358 (1.102-1.674) | 0.004 | 1.180 (0.928-1.500) | 0.176 | 1.794 (1.498-2.150) | <0.001 |
| Log SII tertiles |  |  |  |  |  |  |  |  |
| (0.0000, 3.0297) | Ref. |  | Ref. |  | Ref. |  | Ref. |  |
| (3.0309, 3.5250) | 1.067 (0.843-1.350) | 0.589 | 1.058 (0.791-1.415) | 0.703 | 0.995 (0.712-1.389) | 0.976 | 1.187 (0.929-1.517) | 0.170 |
| (3.5251, 4.6654) | 2.092 (1.685-2.597) | <0.001 | 1.652 (1.252-2.180) | <0.001 | 1.399 (1.008-1.942) | 0.044 | 2.360 (1.881-2.960) | <0.001 |

Model 1 without adjustment; model 2 adjusting anion gap, chloride, WBC, glucose, RDW; model 3 adjusting anion gap, chloride, WBC, glucose, RDW, heart rate, age, SpO_2_, respiratory rate, vasopressor, mechanical ventilation, sex; model 4 adjusting SOFA and GCS scores. SII: systemic immune-inflammatory index, COPD: chronic obstructive pulmonary disease, OR (95% CI): odds ratio (95% confidence interval).
