# Supplementary material for: Relationship Between Systemic Immune‐Inflammation Index and In‐Hospital Mortality in Sepsis Combined With Chronic Obstructive Pulmonary Disease Modified by Mechanical Ventilation
Source: Clin Respir J. 2025 Sep 24;19(9):e70122. doi: 10.1111/crj.70122 (PMC12458911; doi:10.1111/crj.70122)
Supplement: Supplementary file 3 — Table S3: Comparison of SII and other inflammation indicators. [file CRJ-19-e70122-s003.docx]

| **Table S3** Comparison of SII and other inflammation indicators | | | | | |
| --- | --- | --- | --- | --- | --- |
| Variables | AUC | Sensitivity | Specificity | Youden Index | Accuracy |
| NLR | 0.668(0.631-0.706) | 0.615(0.553-0.761) | 0.692(0.541-0.731) | 0.307(0.244-0.374) | 0.665(0.592-0.704) |
| PLR | 0.621(0.581-0.665) | 0.527(0.463-0.689) | 0.707(0.547-0.768) | 0.234(0.184-0.308) | 0.655(0.577-0.702) |
| SII | 0.621(0.577-0.661) | 0.568(0.405-0.666) | 0.674(0.551-0.818) | 0.242(0.179-0.310) | 0.670(0.583-0.730) |

NLR: neutrophil–lymphocyte ratio, PLR: platelet–lymphocyte ratio, SII: systemic immune-inflammatory index, AUC: the area under the curve.
